# Affective Salience Can Reverse the Effects of Stimulus-Driven Salience on Eye Movements in Complex Scenes

**DOI:** 10.3389/fpsyg.2012.00336

**Published:** 2012-09-25

**Authors:** Yaqing Niu, Rebecca M. Todd, A. K. Anderson

**Affiliations:** ^1^Affect and Cognition Laboratory, Department of Psychology, University of TorontoToronto, ON, Canada

**Keywords:** affective salience, visual salience, eye movements, attention, top-down, bottom-up, stimulus-driven, regions of interest

## Abstract

In natural vision both stimulus features and cognitive/affective factors influence an observer’s attention. However, the relationship between stimulus-driven (“bottom-up”) and cognitive/affective (“top-down”) factors remains controversial: Can affective salience counteract strong visual stimulus signals and shift attention allocation irrespective of bottom-up features? Is there any difference between negative and positive scenes in terms of their influence on attention deployment? Here we examined the impact of affective factors on eye movement behavior, to understand the competition between visual stimulus-driven salience and affective salience and how they affect gaze allocation in complex scene viewing. Building on our previous research, we compared predictions generated by a visual salience model with measures indexing participant-identified emotionally meaningful regions of each image. To examine how eye movement behavior differs for negative, positive, and neutral scenes, we examined the influence of affective salience in capturing attention according to emotional valence. Taken together, our results show that affective salience can override stimulus-driven salience and overall emotional valence can determine attention allocation in complex scenes. These findings are consistent with the hypothesis that cognitive/affective factors play a dominant role in active gaze control.

## Introduction

In natural vision human observers sequentially allocate focal attention to sub-regions of a scene (James, 1890). Such attention shifts are typically associated with eye movement behavior (Rizzolatti et al., 1987). Previous research shows that both visual stimulus-driven (“bottom-up”) and cognitive/affective (“top-down”) factors influence the competition for a share of our limited attention (Corbetta and Shulman, 2002).

Bottom-up visual salience models explain guidance of eye movements based on the concept of a visual salience map (Koch and Ullman, 1985; Findlay and Walker, 1999). Shifts of attention and eye movements are initiated toward the point with the highest salience, which is then inhibited so that attention can be disengaged and be moved to the next most salient location. In this way, these visual salience models suggest a control mechanism for dynamically targeting eye movements. These models suggest that low-level feature discontinuities represented in the salience map can explain a significant proportion of where people look. Thus they specify filters that quantify visual conspicuity, a measure of what is perceived as significantly distinct from its local background of each part of the scene.

Computational models have been developed with two types of approaches. The first uses known properties of the visual system to generate a salience map. In these models, the visual properties present in an image generate the visual salience map that explicitly marks regions that are different from their surround such as color, intensity, contrast, and edge orientation (Koch and Ullman, 1985; Itti and Koch, 2000; Parkhurst et al., 2002; Torralba, 2003), contour junctions, termination of edges, stereo disparity, and shading (Koch and Ullman, 1985), and dynamic factors such as motion (Koch and Ullman, 1985; Rosenholtz, 1999). The Itti and Koch (2000) model is frequently cited on behalf of this type of computational visual salience model. A second approach uses scene statistics to determine the relative visual salience of regions of a scene. In this approach local scene patches surrounding fixation points are analyzed to determine whether fixated regions differ in some image properties from regions that are not fixated. For example, high spatial frequency content and edge density have been found to be somewhat greater at fixated than non-fixated locations (Mannan et al., 1996, 1997). Furthermore, local contrast is higher and two-point intensity correlation is lower for fixated scene patches than control patches (Reinagel and Zador, 1999; Krieger et al., 2000; Parkhurst and Niebur, 2003). The spectral residual (SR) method (Hou and Zhang, 2007) is an example of this type of computational visual salience model. It is based on the principle that the human visual system tends to suppress responses to frequently occurring features, while at the same time remaining sensitive to features that deviate from the norm. In a previous study (Niu et al., 2012) we compared the capacity of classical Itti bottom-up model and the SR model in predicting eye fixations. Results confirmed that the SR model does a better job at predicting attention allocation than the classical Itti model.

Yet there is evidence that visual salience does not account for all aspects of a scene that bias attention. For example, semantic meaning and social relevance of elements within a scene also influence allocation of overt attention. A recent study showed that visual salience could not fully account for where observers look within social scenes (Cerf et al., 2008, 2009). Cerf et al. showed that the model that best predicted where observers fixated within scenes was a salience model combined with a face-detection model. This combined-model outperformed the salience model alone. Birmingham et al. (2009a,b) also demonstrated that, when asked to look at a visual scene that includes human faces, participants most frequently fixate on the eyes – a tendency that is not accounted for by computationally modeled bottom-up visual salience. These studies shed light on attentional biases favoring faces and eyes, which cannot be fully explained by the standard bottom-up visual salience models. Thus it is not only visual conspicuity that preferentially commands attention in a complex visual scene.

The affective salience, or motivational importance, of a stimulus may also influence the relatively reflexive allocation of attention. Affective salience engages resources based on the motivational importance of a stimulus in relation to the long-term goals of approaching pleasure or avoiding pain (Todd et al., 2012). Arousal enhanced perceptual learning of salient stimuli but impaired perceptual learning of non-salient stimuli (Lee et al., 2012). Many studies have demonstrated that attention is preferentially allocated to affectively salient relative to neutral stimuli (LaBar et al., 2000; Rosler et al., 2005; Knight et al., 2007). This bias favoring emotional stimuli even occurs under direct instructions to ignore the arousing items (Nummenmaa et al., 2006). Affective salience has also been found to increase viewing duration for both pleasant and unpleasant scenes (Lang et al., 1993) and to capture greater initial attention as well as inhibit subsequent disengagement from a stimulus location (Mogg and Bradley, 1999; Fox et al., 2002). In a recent study, when neutral background scenes were edited to contain a single emotionally salient object and a single visually salient object (Humphrey et al., 2012), more fixations were allocated to affectively salient than visually salient objects. Another recent study found tradeoffs between the influence of visual salience and the reward-punishment value of saccade locations, with value overriding visual salience in attracting saccades at latencies over 184 ms (Schutz et al., 2012). Finally, our own research has revealed that viewers are more likely to fixate emotionally salient than visually salient regions of complex scenes (Niu et al., 2012).

While our previous study demonstrated that observers’ attention to affectively salient regions in a scene is influenced by the emotional valence and arousal of such stimuli, it did not quantify the extent to which the fixations allocated to affectively salient regions are associated with arousal measures. Furthermore, we do not know whether there was a specific bias to look at the affectively salient regions of negatively valenced images. Yet a further question related to the specific stage of visual processing at which emotional factors start to influence eye movement behavior in scene viewing. In summary, no study has examined explicitly the role that emotional salience plays in eye movement behavior. The present study set out to address precisely this issue.

In the present study we measured eye movement fixations during free viewing of negative arousing, positive arousing, and neutral scenes in order to capture the allocation of overt attention during naturalistic scene viewing. Building on previous research, we employed item analysis to investigate the influence of emotional valence and arousal on eye movement behavior within scenes. We hypothesized that: (1) Emotional valence of a scene would influence patterns of attention allocation to salient regions within the scene, and (2) participant arousal ratings for each scene would predict the level of attention allocated to affectively salient relative to visually salient regions within the scene.

## Materials and Methods

### Participants

Participants were 50 young adults (24 female, 18–40 years), with normal or corrected to normal vision and no history of neurological problems, recruited from the University of Toronto campus. Twenty five participants (12 Female) participated in the main eye tracking experiment. Three subjects were excluded from the eye tracking experiment due to eye tracker drifting error, and eye movement data from 22 participants were used. Twenty five participants (12 Female) performed a separate affective salience region of interest generation task. All subjects gave written informed consent for participation.

### Stimulus materials

Twenty five negative and 25 positive photographs were taken from the International Affective Picture System (IAPS). Twenty five neutral photographs were retrieved from the internet as well as the IAPS. Positive and negative images were selected to be similar in overall arousal levels. Positive, negative, and neutral images were equated in log luminance, *F*(2, 72) < 1, and RMS contrast, *F*(2, 72) < 1, which were computed using the Image Processing Toolbox packaged with Matlab 7.0. Positive and negative images were selected to be equivalent in standardized ratings of emotional arousal (emotional salience). Scene complexity and difficulty of figure ground segregation were also rated by a separate set of participants. Participants were asked to rate how difficult it was to discriminate the focal figure of the scene from the background on a scale of 1–7, as well as the composition of each image on from simple to busy or complex on a scale of 1–7. Negative, positive, and neutral images also did not differ in difficulty of figure ground discrimination, *F*(2, 72) < 1, *p* > 0.5, or scene complexity (scale of 1–7), *F*(2, 72) < 1, *p* = 0.5, whether they contained single vs. multiple objects, *F*(2, 72) < 1, or in the number of human figures, *F*(2, 72) < 1, *p* > 0.6.

### Eye tracking experiment

#### Apparatus

Eye movement recoding experiments were programmed in Experiment Builder and analyzed in DataViewer (SR Research). Eye movements were recorded using an infrared eye tracking desktop monocular system – EyeLink 1000 (SR Research, Mississauga, ON, Canada). Stimuli were shown on a 21W ViewSonic G225f monitor positioned 63 cm away from the participant, with a refresh rate of 140 Hz. Participants sat in front of the computer monitor and a chin rest was used to limit head movements. Throughout the experiment, the observer’s right eye position was recorded and sampled at a rate of 1000 Hz. Pictures were presented at a visual angle of 11.17° × 8.37°. We used the manufacturer’s software for calibration, validation, drift-correction, and determining periods of fixation. A nine-point calibration was performed at the start of the experiment followed by a zero-point calibration accuracy test. An additional drift-correction was performed whenever an observer failed to fixate within about 1.4° – (50 pixels) of an initial central fixation cross within 5 s. In all experiments and conditions, each trial started with a central fixation cross which observers had to fixate for 500 ms to trigger stimulus onset.

#### Experimental procedures

After informed consent and a brief practice session, participants performed the free viewing task while eye movements were recorded. Following calibration and validation, participants were shown each of the 75 images in a randomized sequence. Each image was shown for 2 s, and was preceded and succeeded by 2 s of black screen to minimize the possibility of proactive or retroactive interference, making each trial 6 s in length (2 s blank – 2 s stimulus – 2 s blank). Prior to presenting the stimulus, drift-correction was performed to ensure consistency across all trials. Because pilot data indicated that even simple cognitive or memory tasks could alter the participants’ eye movement pattern and fixation compared to a free viewing condition, participants were instructed to view the pictures in a natural manner. To guarantee consistent performance and to maintain concentration throughout the entire testing period (up to 20 min), participants were given two mandatory breaks after the 25th and the 50th trial.

### Affectively salient regions of interest generation task

#### Procedure

In order to generate regions of interest (ROIs) reflecting the most affectively salient regions of each image used in the task, participants were shown each of the 75 photo stimuli in a randomized sequence. For each image, they were instructed to click the mouse in the center of each of the five parts of each picture that were the most emotionally charged in order of intensity (from most intense to least intense). Participants were instructed as follows, “You will be shown a series of images. We want to know which parts of each image you find to be the most emotionally important or arousing. Please click the mouse in the center of the five parts of each picture that are the most emotionally charged for you in order of intensity (from most intense to least intense). This region could be a person or object or a part or combination of either.”

To justify our choice of emotional salience ROIs generation task, we did a pilot study using a different subject-determined emotional salience ROIs task. In the pilot task participants were asked to click as rapidly as possible on the five parts of each image that caught their interest in order of interest. They were instructed to “go with their guts,” and not “over think” their choices. Comparison of the two tasks revealed that the ROIs created by the pilot task were highly correlated with those chosen in the emotional salience task despite different subjects in both studies, suggesting that what is considered interesting is what is most affectively charged and both tasks predicted fixation patterns better than visual saliency maps. In order to precisely predict the *xy* coordinates of fixations without pre-specifying the size or scale of the region that would be chosen, we had participants select a single pixel rather circle whole objects.

**Figure 1:**
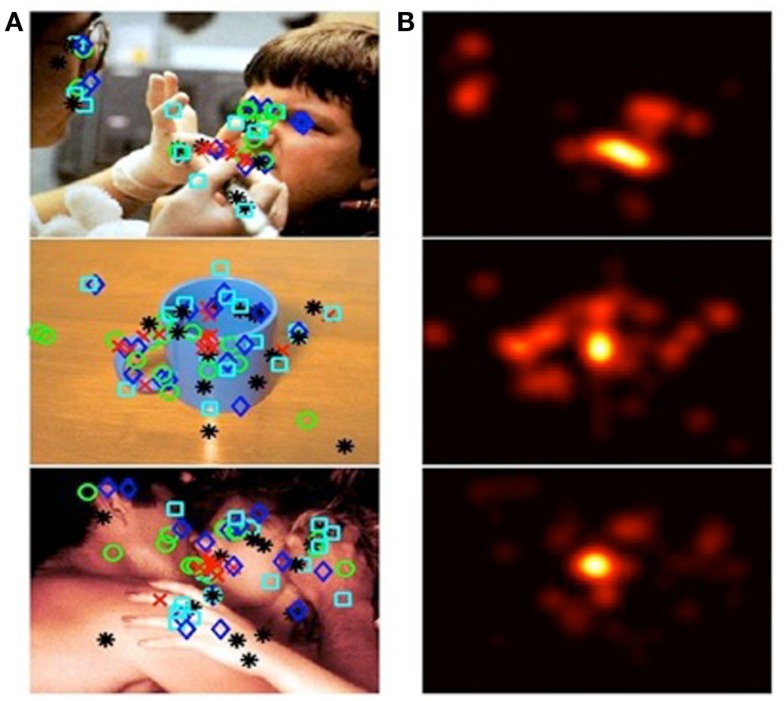
**Generation of affectively salient ROIs**. Column **(A)** from top to bottom, images categorized as: negative, neutral, or positive. Shapes overlying the images denote spots that participants identified, via mouse clicks, as affectively salient. Different shapes denote participants’ order of preference. Column **(B)** affective salience maps, generated from participants’ responses to images in column **(A)**.

The coordinates of the clicked pixel were processed using two-dimensional convolution with a 50-point Gaussian distribution window using Matlab, and an affective salience map representing the average affective salience value across participants was created for each stimulus picture. Then we generated affectively salient regions based on the affective salience map by ensuring that salient regions comprised 10% of the total image as shown in Figure 1. An example of 5 pixels identified by clicking each picture at the center of the region that participants find the most emotionally meaningful is shown in Figure 1A. Figure 1B illustrates the resulting affective salience map. Following the clicking task, participants rated each image for overall affective salience using a numerical scale from 1 (the image was not emotionally arousing) to 7 (the image was extremely emotionally arousing).

#### Computational visual salience model

**Figure 2:**
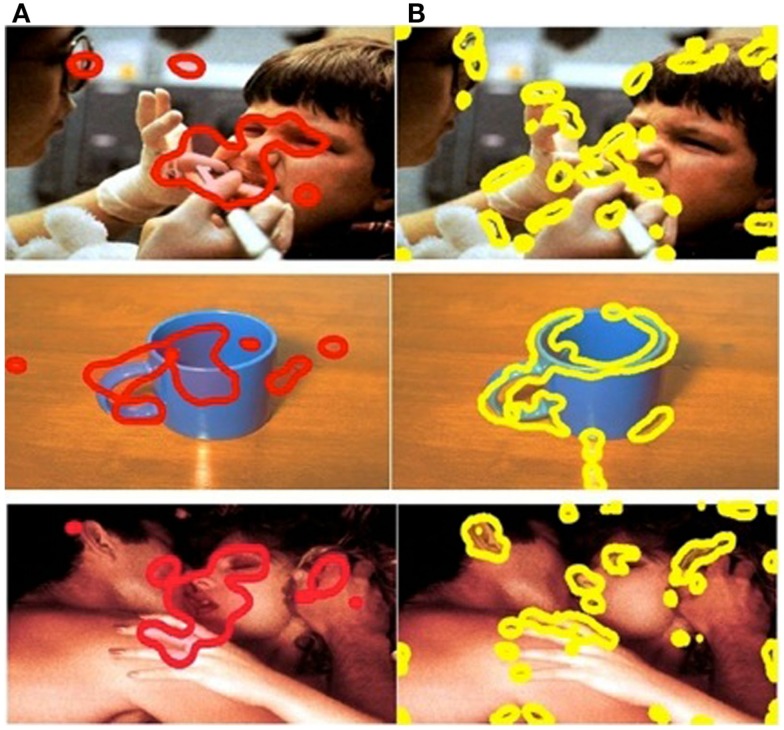
**Comparison of ROIs in example positive, negative, and neutral images**. Column **(A)** affective ROIs. **(B)** Visually salient ROIs.

The SR computational model was implemented to determine the visually salient regions in each stimulus image. The SR model (see Appendix) was adapted by us to detect salient regions. The model was employed to process each image and generated salience maps that visualized salience values. We then generated visually salient regions controlling the coverage of the salient regions (a region with a salience value higher than threshold was considered a salient region; a region with a salience value lower than threshold was considered a non-salient region). The salient regions covered 10% of the total image. The choice of 10% was based on a precedent for object-detection applications used in engineering (Frintrop et al., 2004). This approach allowed us to compare the performance of visual salience and affective salience in predicting eye movement behavior (Figure 2). Affective ROIs are shown in red (Figure 2A) and visual salience ROIs are shown in yellow (Figure 2B).

It is often the case that emotional objects in a scene are also visually salient. We have endeavored to separate these factors by exploring whether emotional regions are still fixated when in competition with other more visually salient regions in the picture. To precisely examine whether emotional salience or visual salience better predicts observed gaze allocation, rather than directly comparing pairs of images or editing the pictures to contain a single emotional stimulus and a single visually salient stimulus, we used methods for emotionally and visually salient region detection within a scene (with emotional salience and visual salience in direct competition). If low-level visual salience is an important factor in attracting attention, then this should still be true when the most visually salient object is not the most emotionally salient one. However, if emotional arousal plays a special role in this attraction, then it could result in the kind of meaning-based override which we have revealed in our previous study (Niu et al., 2012).

#### Eye tracking data

Fixations were calculated using the built-in software of the Eyelink tracking system. A fixation was defined as anything above 70 ms – micro fixations below 70 ms were discarded. We categorized fixations by their “fixation number” based on a fixation’s position in the ordered sequence of fixations (i.e., first, second, third). The “initial fixation” is the fixation occurring before stimulus onset, when the subjects are focusing on the centered fixation cross, and is not counted as part of the ordered sequence of fixations.

Saccades were also determined by the eye tracking system. An eye movement was classified as a saccade when its velocity reached 30°/s or when its acceleration reached 8000°/s^2^. The “saccade planning time” is the duration of time between the stimulus onset and the initiation of the first saccade. Saccade planning times smaller than 50 ms or greater than 600 ms were discarded to remove outliers and artifacts.

The mean number of fixations was calculated for affectively salient and visually salient ROIs to test predictions of eye movement behavior generated by each model. For detailed investigation of eye movement patterns predicted by the emotional category of the image in relation to ROI generated by each model, item analyses were performed examining eye movement behavior image by image for all images used in the task.

## Results

### Item analysis

Previous findings indicated that affectively salient regions overwhelmingly elicited greater attention allocation than visually salient regions (Niu et al., 2012). In order to further explore the influence of emotional valence and arousal on eye movement behavior in ROIs generated by visual vs. affective salience models, we performed item analyses in which we examined eye movement behavior, averaged across participants, for each of the 75 images used in the task. To control for differences in the overall number of fixations between image categories, we calculated the proportion of fixations within each of the affective vs. visual salient regions relative to the number of all fixations in a given image. These fixation allocation tendency scores thus index an increased tendency to fixate in one type of ROI.

**Figure 3:**
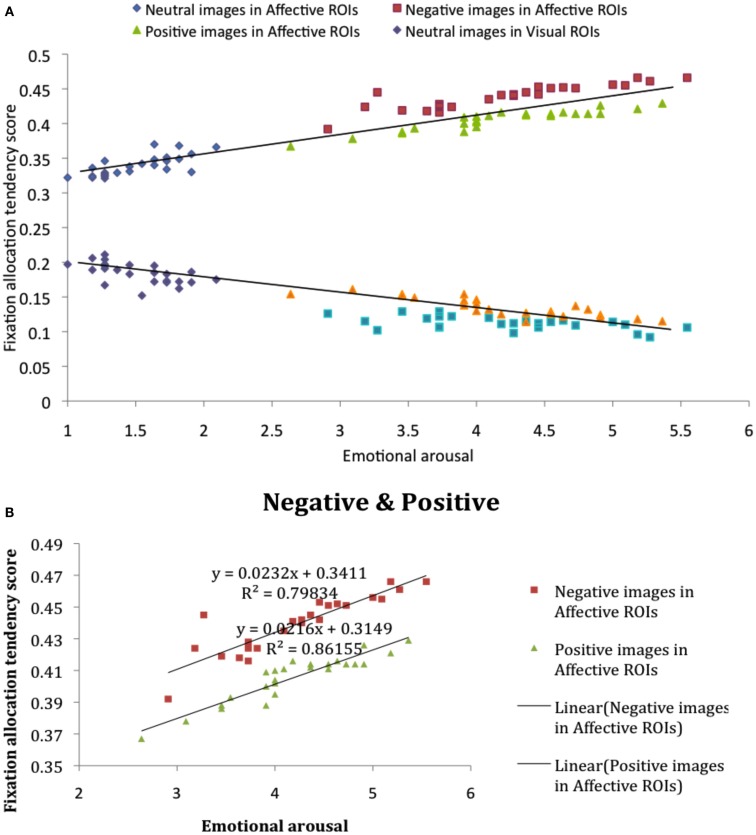
**(A)** Fixation allocation tendency scores in affective and visual ROIs, image by image, as a function of participant-rated emotional salience. **(B)** Fixation allocation tendency scores in affective ROIs for negative and positive image categories.

We first compared fixation allocation tendency scores in affective and visual ROIs, image by image, as a function of participant-rated emotional arousal (Figure 3), based on self-reported arousal ratings for each image (see Materials and Methods). Correlational analysis revealed that, in affective ROIs, fixation allocation tendency scores were positively correlated with arousal (Figure 3A), *R* = 0.93, *p* < 0.001, indicating that participants were more likely to allocate their gaze to affective ROIs when looking at images that were higher in overall arousal. The visual plot of the negative relation between affective salience and allocation tendency scores in Figure 3A, *R* = −0.90, *p* < 0.001, reflects the competition between visual and affective salience regions captured by these tendency scores: An increased proportion of fixations allocated to affective ROIs with increased salience is gained at the expense of fixations to visually salient ROIs. These findings further reveal a stronger effect of negatively valenced stimuli on fixation allocation to affective salience ROIs. Although affective salience was correlated with the proportion of fixations allocated to affective ROIs for both positive and negative images, the intercept for each category of images is markedly different, revealing overall higher fixation allocation tendency scores when viewing negative vs. positive images despite equivalent arousal ratings (Figure 3B).

### Sequence of fixations for affective vs. visual ROIs by emotion category

**Figure 4:**
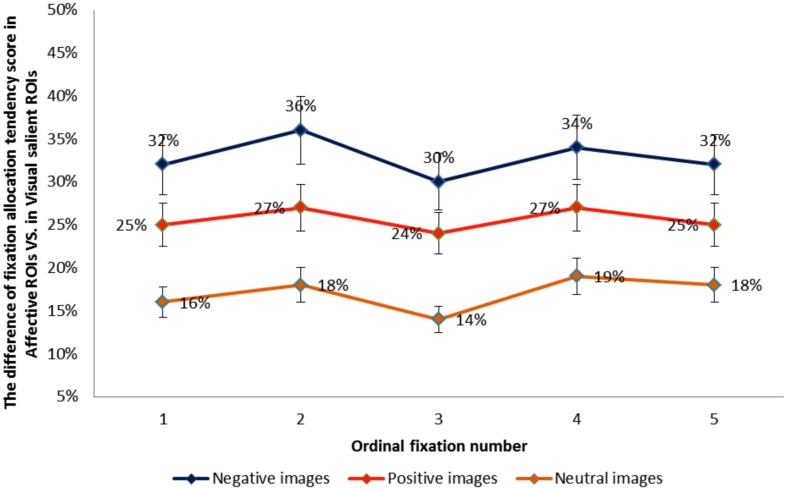
**The difference between fixation allocation tendency scores in the visual vs. affective ROIs for each emotion category as a function of ordinal fixation number**.

We next performed a one-way ANOVA with three emotion category on difference scores between the proportion of fixations allocated to each type of ROI (affective salience > visual salience). Results revealed that the difference between the proportion of fixations allocated to affectively vs. visually salient ROIs was greatest for negative and smallest for neutral images, *F*(2, 72) = 276.45, *p* < 0.001, η^2^ = 0.88. Pairwise contrasts showed that for each emotion category ROI difference scores differed from the other two categories (*p*’s < 0.001). In order to further compare the influence of emotion category on sequential looking order in affectively vs. visually salient ROIs, we created difference scores between fixation allocation tendency scores for the first through the fifth fixation in each type of ROI for each emotion category. Figure 4 illustrates the difference between fixation allocation tendency scores in the two ROIs as a function of ordinal fixation number, showing that the influence of emotion category on the difference in fixations allocated to each type of ROI remains constant across sequential fixations. The results show that the difference in the proportion of fixations allocated to affective vs. visual salience ROIs was greatest for negative images and smallest for neutral images – and that this pattern of results remained constant from the first to the fifth sequential eye movement, which suggests that the emotional factor influences early on in scene viewing.

### Extended analysis of example images

**Figure 5:**
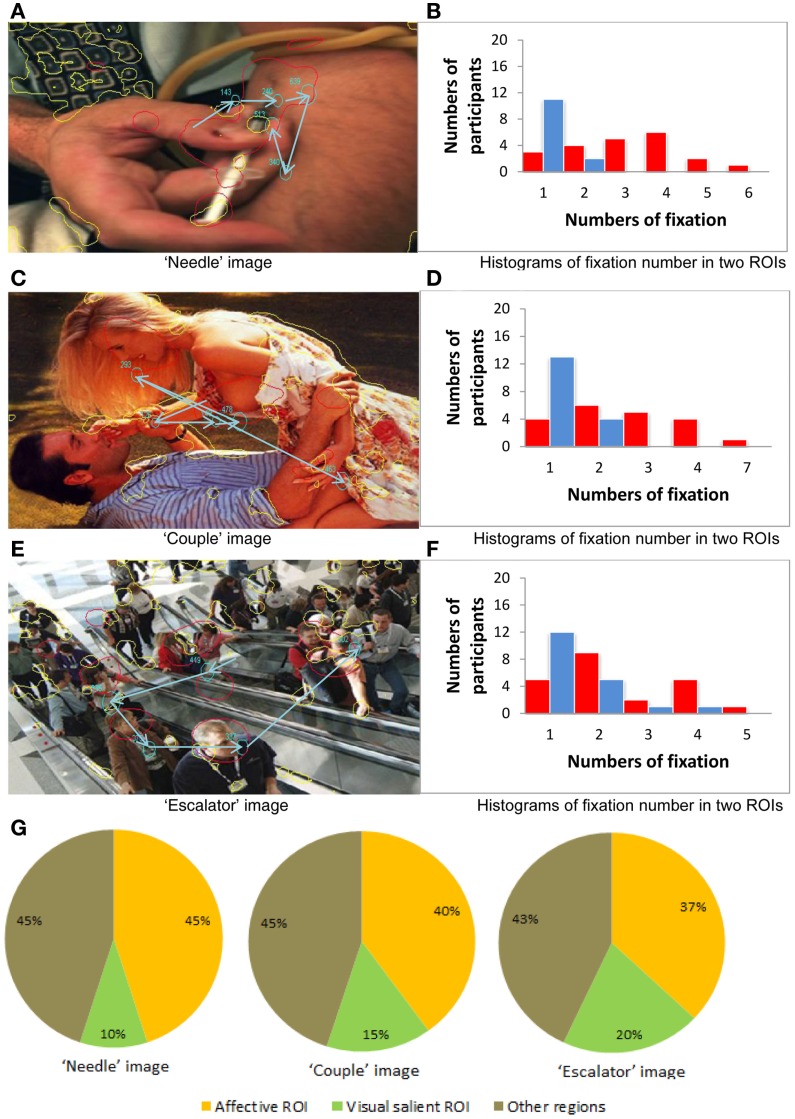
**Eye movement behavior analysis of the three example images**. **(A,C,E)** Three example images. **(B,D,F)** The number of participants with 1–7 fixations in affective vs. visual salient ROIs for each of the example images. **(G)** Fixation allocation tendency scores across all participants for the example images.

Finally, for a more fine-grained examination of fixation allocation when emotional salience and visual salience are in direct competition, we focused on an example image from each image category, choosing three images where there was the least amount of overlap between the two types of ROIs. First, we investigated eye movement behavior, participant by participant, for each of the three example images. Plots in Figures 5B,D,F show the number of participants with 1–7 fixations in affective vs. visual salient ROIs for each of the example images. These plots illustrate the strikingly higher number of fixations allocated to affective over visually salient ROIs when there is minimal overlap between the regions.

For the “needle” image (negative stimulus case), in Figure 5A the red curves illustrate the affectively salient regions and the yellow curves illustrate the visually salient regions. Here we show data from one of the participants whose eye movement scan path in is depicted in blue. Note that the size of the circle denotes the fixation duration and the arrow illustrates sequences of fixations. Figure 5B illustrates the greater number of participants with 1–6 fixations in affective vs. visual salience ROIs, revealing the advantage for affective ROIs for this image.

For the “couple” image (positive stimulus case), we again see in Figure 5C the affectively and visually salient regions, as well as the scan path of one of the participants. In Figure 5D we can still observe a greater number of fixations allocated in affective than in visually salient ROIs (although it is less pronounced than in the negative stimuli case Figure 5B).

For the “escalator” image (neutral stimulus case) the emotionally and visually salient regions are illustrated in Figure 5E. From Figure 5F we can observe a marginally greater number of fixations allocated in affective than in visually salient ROIs.

We next generated fixation allocation tendency scores across all participants for the example negative, positive, and neutral images, as shown in Figure 5G. These pie charts further illustrate the finding we report from the previous item analysis of all 75 images: When visual and affective salience compete, participants are most likely to allocate fixations to affectively salient ROIs in images with an overall negative valence in the absence of overlap between visual and affective salience.

#### Summary

Results of the item analysis revealed that, image by image, the proportion of fixations allocated to affective relative to visual salience ROIs was strongly associated with higher ratings of arousal: Viewing more arousing stimuli increased the likelihood of fixating in emotionally salient regions. This was true of both positive and negative images. Moreover, the difference in the proportion of fixations allocated to affective vs. visual salience ROIs was greatest for negative images and smallest for neutral images. This pattern of results remained constant from the first to the fifth sequential eye movement, suggesting patterns of attention allocation are modulated by the salience of the image early on. Finally, analysis three employed individual participants’ data to examine looking patterns for three example images where competition between visual and affective salience was greatest. This analysis illustrated the findings that were typical across the entire image set in conditions of maximum competition between visual and affective salience: Participants showed more fixations in affective ROIs when looking at each of those images, but the largest proportion of fixations was allocated to affective salience regions in the image with an overall negative valence.

## Discussion

Our results showed that, when participants freely viewed complex scenes, the proportion of fixations allocated to affective relative to visual salience ROIs was associated with higher ratings of emotional arousal, such that viewing emotionally arousing stimuli increased the likelihood of fixating in emotionally salient regions. Yet although the relationship between arousal and likelihood of fixating in affectively salient regions was similar for both negative and positive images, there was an overall higher proportion of fixations allocated to affective ROIs in images which had an overall negative valence. Thus, viewing negatively valenced scenes has an even stronger impact on allocation of overt attention to affective ROIs compared to scenes that are equally arousing but positively valenced, suggesting that our attention to emotive regions in a scene is influenced by the valence of such stimuli. These findings build on previous results showing that participants allocated more eye movements to regions of a given scene that were identified as affectively salient than regions identified as visually salient, particularly for negatively valenced scenes (Niu et al., 2012).

Like previous studies examining the role of semantic/affective salience, we examined number of fixations as a measure of foveal sampling of ROIs in each image. Distinct patterns of overt attention have been previously observed for emotional scenes, with higher fixation counts, or greater sampling of the image space, for arousing vs. neutral scenes (Sharot et al., 2008; Riggs et al., 2010), suggesting that scenes that are globally more arousing elicit more sampling of sub-regions of the image. We have extended such findings to show increased sampling for arousing images in sub-regions of an image identified as more affectively salient.

Taken with our previous findings (Niu et al., 2012), our results indicate that visual salience does have an effect on eye movements when one is inspecting an emotionally arousing scene, but the capacity of affective salience to override visual salience can be plausibly observed.

Previous studies have shown that low-level visual salience helps guide eye movements in free viewing (Parkhurst et al., 2002; Parkhurst and Niebur, 2003, 2004). Yet it is not only visual conspicuity that can produce a pop-out effect in the inspection of an image. There is also evidence that higher-level aspects of a stimulus, such as semantic meaning, can bias attention in favor of socially relevant stimuli (Birmingham et al., 2009a,b; Cerf et al., 2009). When semantic meaning is further associated with emotional arousal, commonly feared, or pleasant stimuli (e.g., a murder scene, erotica) can prioritize attention relative to neutral stimuli (LaBar et al., 2000; Nummenmaa et al., 2006, 2009). Only two other studies to date have examined the competition between visual salience and affective salience within a single complex scene: One study found that, when neutral background pictures were edited to contain a single affectively salient and a single visually salient object, fixations were more likely to be on affectively salient objects (Humphrey et al., 2012).

Our results showed a greater likelihood of fixating on affectively salient regions within negative relative to positive scenes. This finding suggests that negatively valenced scenes have an overall stronger impact on attention allocation to affectively salient regions compared to scenes that are equally arousing but positively valenced. Thus, our attention to emotive regions in a scene is influenced by the valence of such stimuli. At the behavioral level, this effect can be interpreted in the light of previous findings from our lab that negative, but not positive, affect enhances selective visual attention (Rowe et al., 2007; Schmitz et al., 2009). Here, it is possible that negative affect generated by the negative arousing images increased selective attention in a form of “weapon focus” on the most affectively salient items in the scene. At the neural level, the influence of a scene’s overall valence even on early fixations may be supported by rapid responses to valence that have been demonstrated in the orbitofrontal cortex (OFC; Kawasaki et al., 2001). The OFC is reciprocally connected to temporal regions of the visual cortex (Rempel-Clower and Barbas, 2000), which in turn are connected with the lateral intraparietal cortex (LIP) which is important for allocating overt attention (Blatt et al., 1990; Thompson and Bichot, 2005; Goldberg et al., 2006). Such rapid processing of valence information may contribute to subsequent eye movement planning through LIP integration of either direct or indirect information from the OFC.

It has been suggested that the LIP in functions as a priority map that guides attention based on the moment to moment behavioral priority of aspects of the world (Bisley and Goldberg, 2010). By integrating information from other brain regions, including dorsal and ventral streams of the visual cortex, the anterior cingulate cortex, and regions of the thalamus (Blatt et al., 1990; Baizer et al., 1991, 1993), the LIP has been found to influence attention based on bottom-up visual salience, task-related goals, the expected reward value (including social rewards), and the behavioral relevance of a stimulus (Dorris and Glimcher, 2004; Sugrue et al., 2004; Balan and Gottlieb, 2006). Given considerable overlap between the constructs of behavioral relevance and motivational or affective salience, and given LIP connectivity with regions (e.g., the pulvinar nucleus of thalamus) implicated in affective salience tagging (Pessoa and Adolphs, 2010), the LIP may also play a role in prioritizing attention based on affective salience. The amygdala, which along with the pulvinar has been characterized as a motivational/affective salience detector (Cunningham et al., 2008; Todd and Anderson, 2009; Pessoa and Adolphs, 2010), is densely interconnected with multiple regions of visual cortex as well as well as with thalamic nuclei (Amaral et al., 2003; Shipp, 2003). Thus, the LIP may integrate information from the amygdala either directly or indirectly via other brain regions to integrate information about affective salience into a priority map for determining saccades.

Some limitations to the study qualify our interpretation of the results. First, it should be noted that, whereas the visual salience model was computer-generated the ROI in the affective salience model were based on human ratings. Thus, the findings reported here may be influenced by the difference between human and computer-generated models. Second, there was greater similarity in content between images of erotica within the positive category in comparison to between images in the neutral and negative categories. Although affective salience relates to subjective impressions elicited by emotion rather than image categories, the fact that there was greater similarity between images in the positive category than in the negative and neutral categories may have influenced the results. Finally, future studies using human-generated affective salience ROIs should measure the reliability and validity of the affective salience ROI generation task, in particularly for the neutral images where rating consistency may be expected to be lower.

Despite significant recent progress, the best available computational visual salience models still lag behind human performance in predicting eye fixations in free viewing of complex scenes. The majority of models are based on low-level visual features and the importance of top-down factors has not yet been fully explored or modeled. Exploration of a cognition-based computational salience model that integrates semantic meaning and affective salience is an important future research direction. There are a number of applications that would benefit from such research. For example, selective rendering in computer graphics could benefit from improvements on eye gaze prediction models.

In conclusion, our results add to the literature about the influence of emotion on cognition by showing that the affective salience of an object – which can be defined by one’s previous experience with it in relation to overall motivational goals of maximizing pleasure and avoiding pain (Todd et al., 2012) – can influence allocation of attention. They suggest that the overall emotional salience of an image determines allocation of attention to affectively salient regions of a scene, particularly for negative images. Thus, the affective importance of context can prioritize our attention to specific features of the world that are linked to associations between semantic meaning and emotional arousal. Whether this enhances or impairs cognition may depend on the other goals that are active at the time.

## Conflict of Interest Statement

The authors declare that the research was conducted in the absence of any commercial or financial relationships that could be construed as a potential conflict of interest.

## Appendix

### Spectral residual model

It was discovered that an image’s Spectral Residual (SR) of the log amplitude spectrum represented its innovation (Hou and Zhang, 2007). By using the exponential of SR instead of the original amplitude spectrum, the reconstruction of the image results in the salience map. The salience estimation is carried out using this computational model.

$$\begin{array}{llll} A\left(f\right) & =ℜ\left(𝔉\left[J\left(x\right)\right]\right) & & \text{(1)}\\ P\left(f\right) & =𝔍\left(𝔉\left[J\left(x\right)\right]\right) & & \text{(2)}\\ L\left(f\right) & =\log\left(A\left(f\right)\right) & & \text{(3)}\\ R\left(f\right) & =L\left(f\right)-h_{n}\left(f\right)*L\left(f\right) & & \text{(4)}\\ S\left(x\right) & =\text{g}\left(x\right)*{𝔉}^{-1}{\left[\text{exp}\left(R\left(f\right)+P\left(f\right)\right)\right]}^{2} & & \text{(5)} \end{array}$$

In this computational model, the SR R(f) contains the innovation of an image which can be obtained by (4), where L(f) denotes the logarithm of amplitude spectrum A(f) of the image J(f) computed by (3) and *h_n_*(*f*) is the average filter. Using inverse Fourier transform then squared, the salience map in spatial domain is constructed. For better visual effects, we smoothed the salience map S(x) with a Gaussian Filter *g*(*x*) as (5), where $F\text{ and }F^{-\text{1}}$ denote the Fourier transform and inverse Fourier transform, and P(f) denotes the phase spectrum of the image.
